# Pulp Protection by Cavity Lining

**Published:** 1892-08

**Authors:** G. F. Cheney

**Affiliations:** St. Johnsbury, Vt.


### Pulp Protection by Cavity Lining.

BY G. F. CHENEY, D.D S., ST. JOHNSBURY, VT.

Read before the Vermont State Dental Society, March, 1892.

In presenting the subject of pulp protection I aim rather to bring out a discussion of a much-neglected subject than to offer anything particularly new (I am sure most of us see evidences of this neglect in our daily practice), neither do I intend to discuss pulp capping, but protection of unexposed pulps from thermal influence through metallic fillings by cavity lining.

It is a well-known fact that thermal influence will account for the death of thousands of pulps under gold and amalgam fillings, and this is the beginning of a series of disturbances which in time result in abscesses.

I have seen so many abscessed teeth, where, upon removal of the filling, sufficient apparently sound tissue was found to have fully protected the pulp, that I am inclined to believe that in this climate some protection should be used under most of our metallic fillings, especially in approximal cavities of lateral incisors and bicuspids where the pulps come so near the surface. Without doubt, if a properly inserted lining had been used in these cavities the pulps could not have been anything but preserved. Of course it is possible that in a few instances the death of the pulp may have been caused by violence, like a blow, or in biting hard substances.

In teeth where there is the slightest chance for the pulp to become injured by thermal or electric influences the safe course is best. Too much thought and care cannot be given to the protection of the pulp. We must always bear in mind the larger proportional size of the pulp in early life, the possibility of its occupying an abnormal position, the chances of there being a crack or fissure extending to it, and of a point of it coming nearly to the surface. (These chances are very beautifully illustrated in the *International Dental Journal* by Dr. Andrews.)

We must also be able to diagnose the difference between sensitive dentine and tenderness of the pulp. Sensitive dentine responds when excavating over a considerable portion of the cavity walls, but does not respond to simple pressure; while, when in near proximity to the pulp it responds quite as readily to pressure as to the cutting instrument and is confined so completely to a single point that the danger is at once suggested to the operator.

In deep-seated cavities the necessity of caution becomes greater and the danger of intruding upon pulp territory increases, and unless carefully protected thermal changes may prove a disturbing influence which will give rise to more serious trouble.

For the protection of the pulp against these influences I should recommend something with as little conductivity as the case will admit. Scores of different materials have been in use; of these I will only mention a few, namely: varnish and the various zinc plastics, oxyphosphate, oxysulphate and oxychloride of zinc. To me varnish is the most useful. I use the sandarac gum dissolved in alcohol, and quite thin. The effect of varnish lining is to leave upon the cavity walls a thin semi-opaque whitish film which is non-conducting, non-irritating, insoluble and more in harmony with dentine than any metallic substance, and can be used in any cavity no matter how shallow, because of the small amount of space it occupies. The operation of varnish-lining is very simple, having the rubber-dam adjusted and the cavity properly dried, a small pellet of cotton is dipped in the varnish, conveyed to the cavity touching the bottom and walls. Five or ten minutes should be allowed for hardening which can be hastened by hot air. In some cases I take a piece of tissue paper, dip in the varnish and place over the cavity bottom.

In approximal cavities of the posterior teeth, especially those extending below the gum margin, we sometimes find ourselves in close proximity to the pulp, with barely depth enough for anchorage to the filling, I find nothing else will take the place of varnish in these cases for a lining. Varnish will prevent filling material showing through thin enamel walls, which might be very unsightly without it.

Oxyphosphate of zinc is an excellent liner, it is adhesive, does not shrink, and is indicated where the walls of the tooth need strengthening. In deep-seated cavities where undercuts exist, if the enamel is strong it need not be cut away, for when hard cement is carefully packed in its place it forms a support when hard, almost equivalent to dentine. A cavity cut in it to a depth a little greater than enamel, reduces the final filling with gold to an operation of the simplest character, as this cavity has a hard, firm base of cement and a boundary of cement and tooth substance, or of the latter alone. When using an oxyphosphate in deep-seated cavities we must not forget the necessity of protecting the pulp against the effect of phosphoric acid. This can be done by varnishing the bottom, or by using a little oxyphosphate of zinc, or a part of oxide of zinc and oil of cloves.

Oxysulphate of zinc is probably one of the best pulp protectors which has ever been used, is easy of adaptation and perfectly non-irritating, alike to sensitive dentine and to the dental pulp, and is probably the best material to be used where the pulp is nearly exposed. Place a small amount of the thinly mixed oxysulphate over the bottom of the cavity, allowing a few minutes to set, then finish the lining by covering it with oxyphosphate of zinc.

Oxychloride of zinc, although used quite extensively, is not a reliable liner except when used in small quantity, it being a notable shrinker when used in bulk, which makes it very much inferior to the phosphates when we wish a strengthener for the cavity walls. It is irritating and should not be used near the pulp, except over varnish or oxyphosphate. It has been said that oxychloride of zinc permits of no decay in adjoining tooth structure.

In 1888 Dr. Kells, of New Orleans, by the use of the thermostat, an electric instrument, demonstrated before the joint meeting of the American and Southern Dental Societies, at Louisville, Ky., the conductivity of heat and cold through filling material.

He says: These may be divided into classes, the metals coming first as the best conductors, the difference between them being slight; next comes the cements, the oxyposphates being a shade poorer than the oxychlorides; then the gutta-perchas come last, although far from being non-conductors, not even equalling enamel. He further says that the oxyphosphates and oxychlorides are such comparatively good conductors of heat and cold, that they should not be used alone for capping pulps, exposed or nearly exposed. That such pulps should be protected by a layer of gutta-percha fully 1-16th of an inch in thickness when possible.

Gutta-percha I have used very little and hardly feel like saying much about it, but from what little experience I have had with it, I should be afraid to use it near the pulp in such quantity as Dr. Kells recommends for fear of expansion; would rather depend upon varnish.

In a paper read before the First District Dental Society of New York last October by Dr. Kells, he further says: The method of testing the thermal conducting powers of various filling materials, and comparing them in that respect to enamel, is as follows: The enamel of a sound molar was hollowed out into the shape of a cell. Similar cells of the same size were made of gold, tin, amalgam, oxyphosphate of zinc, oxychloride of zinc, red gutta-percha and Hill's stopping. In the apparatus before me this thermostat is connected, the cells of the materials to be tested, and warm water is ready for demonstrating their conductivity. I will first place upon the disk the cell of gold, and filling it with warm water, our ears at once corroborate what we already know as to its conductivity. For no sooner had the gold cell been filled with the water than it at once conducted the heat to the disk beneath. Replacing the gold by tin and amalgam successively the same results are produced.

The oxychlorides and oxyphosphates will follow, when it is readily perceived that more time is required to heat the disk, thus demonstrating them to be poorer conducting agents than the metals, but far from being non-conductors.

We will next try gutta-percha and Hill's stopping, which prove themselves much poorer conductors; but give them time, and the bell does ring.

Lastly, I place the enamel cell on for demonstration, with the result that sufficient heat to close the circuit is not transmitted to the disk.

From these experiments we may conclude that, contrary to the general opinion held, the oxyphosphates and the oxychlorides of zinc are relatively good conductors of thermal effects, and further, that for protecting the dental pulp nature has provided a shield that has not yet been successfully replaced by dental science.

One other combination I wish to speak of, although perhaps not strictly belonging to the subject.

Some four or five years ago I filled a bicuspid approximally with amalgam, not thinking of any possible chance of thermal trouble. A few days later the patient returned, saying that she could not take the cold water or breathe cold air into her mouth without pain in the tooth. I tried several remedies, but to no avail; the trouble still continued. Finally I drilled into the crown, making a small cavity, which I filled with gold in such a manner that the gold came in contact with the dentine of the tooth and the amalgam filling, when the trouble ceased. Since then I have treated one or two other cases in the same manner with equally good results. In a paper read by Dr. Stockwell before the Odontological Society of New York, he explains this action thus: In regard to the question of thermal influences there is no doubt but that Robinson's foil is a better non-conductor than gold, and this would in part at least account for the immediate favorable results. But there can also be little doubt that the galvanic action set up by this combination taken in connection with the fluids of the mouth, lends an impulse toward the removal of those physiological sensations resulting from thermal influences. There can be no doubt at all that the combination of amalgam and gold, when placed in contact with the teeth and fluids of the mouth, will create an electric current, and I am assured by competent authority that when a tooth is so filled the current will flow in the following direction, namely, from the amalgam down through the body of the tooth to the pericementum, from it to the saliva, and from it or through it to the gold.

In the *Dental Review* for February, this year, are given some experiments upon the conducting powers of different materials which are used for filling purposes by Dr. Gilmer, of Chicago, as follows: Gold, 1000; Lawrence amalgam, $852\frac{5}{11}$; copper amalgam, $702\frac{7}{10}$; tin, 590; oxyphosphate of zinc, $584\frac{27}{110}$; oxychloride of zinc, $525\frac{25}{100}$; artificial dentine, 525; gutta-percha, 520. He also states: A test of the conducting qualities of alloys presents curious results. For instance, if one per cent. of silver, which is represented as a conductor by 1000, be added to gold, the conducting of the alloy is changed from 980, which gold alone gives, to 840. If two metals be combined, one being the best known conductor, and the other the poorest, the latter predominating, the conducting quality of the alloy formed is no better than if it did not contain a particle of the better conductor. All this proves to my mind that combination fillings of gold and tin, or gold and amalgam, should be classed among the pulp protectors.

